# From Mysterious Supernatant Entity to miRNA-150 in Antigen-Specific Exosomes: a History of Hapten-Specific T Suppressor Factor

**DOI:** 10.1007/s00005-015-0331-4

**Published:** 2015-02-18

**Authors:** Włodzimierz Ptak, Katarzyna Nazimek, Philip W. Askenase, Krzysztof Bryniarski

**Affiliations:** 1 Department of Immunology, Jagiellonian University Medical College, ul. Czysta 18, 31-121 Kraków, Poland; 2 Department of Internal Medicine, Yale School of Medicine, 333 Cedar St., New Haven, CT 06520 USA

**Keywords:** T suppressor factor, T suppressor cells, miRNA, Exosomes, Immune suppression, Immune tolerance, Tolerosomes

## Abstract

Soon after the discovery of T suppressor cells by Gershon in 1970, it was demonstrated that one subpopulation of these lymphocytes induced by i.v. hapten injection suppresses contact sensitivity response mediated by effector CD4+ or CD8+ T cells in mice through the release of soluble T suppressor factor (TsF) that acts antigen specifically. Our experiments showed that biologically active TsF is a complex entity consisting of two subfactors, one antigen specific and other non-specific, produced by differently induced populations of cells. In following years, we found that the antigen-specific subfactor is a light chain of IgM antibody that is produced by B1a lymphocytes. However, the exact nature of non-specific part remained a mystery for about 30 years. Our current studies characterized TsF as regulatory miRNA-150 carried by T suppressor cell-derived exosomes that are antigen specific due to a surface coat of IgM antibody light chains produced by B1a cells. The present communication briefly summarizes our studies on TsF that led to discovery of regulating miRNA that acts antigen specifically to suppress immune response.

## Introduction

Adaptive immune responses can be classified as cell-mediated immunity (CMI), in which antigen-specific T lymphocytes are the effector cells, or humoral immunity mediated by antibodies. CMI reactions in contrast to humoral immunity can be transferred from immunized donors to naive syngeneic recipient by lymphoid cells but not by serum. CMI reactions encompass such phenomena as immunity to many viral and bacterial infections, graft rejection, graft versus host reaction and some forms of autoimmune responses and allergic reactions of type IV. These are represented by delayed-type hypersensitivity (DTH, like tuberculin reaction) or contact sensitivity (CS) reactions (Askenase 2014). Both can be elicited by antigen in the skin of immunized subjects and clinical manifestations (local inflammatory reaction) appear hours or even days after antigen recognition by effector cells. While classical DTH responses can be elicited by protein antigens, CS is an inflammatory response induced by simple chemical compounds (haptens) applied on the skin, that become immunogenic after conjugation with skin self-proteins (neo-antigen). Dermal antigen-presenting cells (Langerhans cells and other dendritic cells) carry these neo-antigens to regional lymph nodes where they are recognized in the context of class II or I of MHC by precursors of effector CD4+ or CD8+ T cells, respectively (Kaplan et al. 2012). Immune T cells then migrate to the site of antigen deposition in the skin to mount the local inflammatory response. CS responses remain under the control of regulatory mechanisms encompassing down-regulatory (suppression) and up-regulatory (contrasuppression) circuits (Green and Ptak 1986; Ptak et al. 2009).

This short article summarizes experiments performed during years since the original work of the 1970s in three closely cooperating laboratories (Medical College of Jagiellonian University, Cracow, Poland and Departments of Pathology and Clinical Immunology at Yale University, New Haven, CT, USA) on the mechanisms of induction of suppressor factors produced by suppressor cells and on the mechanisms of their action by which they inhibit in antigen-specific manner the activity of immune cells that mediate contact sensitivity reactions in mice. The results are arranged not necessarily in chronological order as they were published.

### Passive Transfer of Contact Sensitivity

Initially, studies on mechanisms of DTH reactions were performed in guinea pigs. These animals served as a good model for testing immune response in the skin, since the symptoms of inflammation were clearly visible and measurable. Landsteiner and Chase (1942) studied the immune response to simple chemical compounds and demonstrated the experimental transfer of CS reaction into naive guinea pigs through the intravenous (i.v.) injection of peritoneal exudate cells (PEC) from immunized donors. Subsequently, Chase (1945) was also able to passively transfer DTH reaction to protein antigen (tuberculin) with PEC and lymph node and spleen cells into inbred guinea pigs. These experiments allowed to study the mechanisms of DTH response and their regulation as well. Nevertheless, the most challenging disadvantages of using guinea pigs were their low reproductiveness as well as shortage of inbred strains. The first attempts to induce DTH reactions to protein antigen (like tuberculin) or contact sensitivity in mice and to transfer of the response into syngeneic recipients were only partially successful mainly due to the standard testing procedures used (Crowle 1960; Crowle and Crowle 1961). As compared with guinea pigs, in which elicited DTH or CS reactions at the flank skin are easily visible and measurable, the thickness of mouse skin and, as later shown, paucity of skin mast cells allows only for insufficient swelling and other signs (like reddening) (Gershon et al. 1975; Kops et al. 1984). Asherson and Ptak (1968; Ptak and Asherson 1969) were the first who induced CS to picryl chloride (PCL), oxazolone (OX) and dinitrofluorobenzene (DNFB) in mice and transferred this reaction by i.v. administration of immune cells (PEC or lymph node and spleen cells) into syngeneic animals but not in outbred mice. They also proposed a new method for measurement of CS with engineer’s micrometer by estimation in actively and adoptively sensitized animals of increase of thickness of ear, on which immunizing hapten was deposited (apart from hind limbs, mouse ear is a single place containing rich concentrations of cutaneous mast cells). This method allowed the future study on mechanism of development and regulation of CS. Later Ptak and Jaszcz (1969) characterized the CS lesions induced by oxazolone in the mouse ear histologically as containing principally neutrophils, which was later confirmed for PCL by Roupe and Ridell (1979). Simultaneously we made, as turned out later, an important observation that, although the peak of CS reaction as measured by ear swelling culminates at 18–24 h after hapten application, there is also an early transient swelling reaction at approximately 2–4 h, which then subsides (Asherson and Ptak 1968; van Loveren et al. 1983).

### Immunological Unresponsiveness and Suppression

Immunological unresponsiveness was firstly observed by Sulzberger (1929) in guinea pigs as an effect of antigen administration through non-classical route (i.v. instead of epidermal). While epicutaneous route led to activation of immune response to this antigen, animals pretreated by i.v. route did not become sensitized when subsequently immunized epidermally. Later Chase and co-workers (Battisto and Chase 1955; Chase 1946, 1967; Chase and Maguire 1973) and others (Pomeranz 1970) found that also feeding of guinea pigs with antigen or hapten induced state of immunological unresponsiveness (Sulzberger-Chase phenomenon). Unresponsiveness was also produced in guinea pigs by either i.v. or oral repeated administration of free hapten as well as i.v. injection of hapten conjugated blood lymphoid cells or splenocytes, cell membranes and proteins (Battisto and Bloom 1966a, b). Initially the most commonly used method of inducing unresponsiveness in mice was the i.v. injection of water soluble form of hapten (e.g., trinitrobenzene sulphonic acid—TNBSA in case of PCL hapten). The induced unresponsiveness was antigen-specific, i.e. animals made tolerant to TNP were fully responsive to OX and vice versa. However, after i.v. injection of TNBSA skin of mice remained yellow for over a week (Asherson and Ptak 1968), which suggested that hapten was bound to various proteins and cells throughout the body and, therefore, might interfere with other elements of immune response. Later, Fidler and Golub (1973) have shown that i.v. injection of free hapten results in development of unresponsiveness of both T and B cells. To test this assumption, we injected mice intravenously with TNP-conjugated cells (mouse erythrocytes or lymphocytes) or TNP-labeled murine immunoglobulins and then tested for CS to PCL and for antibody production to TNP using TNP-labeled sheep erythrocytes as antigen (Ptak and Rozycka 1977). The results showed that each procedure produces split tolerance. Thus, mice that received TNP-labeled cells were tolerant to skin sensitization but production of anti-TNP antibodies remained unaffected, while injection of TNP-immunoglobulins made mice tolerant in the antibody humoral compartment, leaving CS reactivity untouched. Thus, in further experiments to produce unresponsiveness affecting only CS we used hapten-labeled mouse erythrocytes or thymocytes as tolerogen. Parenthetically, even skin painting with PCL, which leads to development of CS, simultaneously produces some level of tolerance in the humoral compartment because of leaking of TNP into circulation, which results in labeling of blood proteins (Rozycka and Ptak 1978).

### Early Studies on T Suppressor Cells and T Suppressor Factor in CS

Asherson and Ptak (1970) found that CS to picryl chloride (PCL) was depressed by pretreatment of mice with the sulphonic acid derivative of PCL. They used these mice and their X-irradiated (850r) counterparts to study the restoration of immune competence (i.e., the ability to develop CS reactions upon sensitization) after i.v. transfer of normal lymphoid cells. No restoration was observed when non-irradiated recipients were used. In contrast, X-irradiated mice supplemented with the mixture of lymph node and bone marrow cells from normal donors were able to mount CS reaction at the normal level upon sensitization. In view of the earlier discovery of T suppressor cells by Gershon (Gershon and Kondo 1970, 1971; Gershon et al. 1972), it seemed reasonable to assume that X-irradiation removed a blockade (i.e., possible suppressor cells) from the body of tolerized animals that prevented the immunization of transferred normal cells. In a following series of experiments Zembala and Asherson (1973) demonstrated that indeed cells from TNP tolerized mice admixed with immune cells from actively sensitized animals prevented their successful transfer of CS into naive mice. They also tested whether lymphoid cells from mice made tolerant by i.v. hapten administration would produce upon in vitro culture an inhibitory product that could replace suppression by the cells. It was shown, however, that these supernatants had no suppressive activity and the suppressive effect (inhibition of adoptive transfer of CS effector cells) was only achieved when Ts cells were from mice, in which i.v. tolerization by hapten was followed by skin contact sensitization with the same hapten (Asherson and Zembala 1974a; Zembala and Asherson 1974). The active moiety present in the supernatant was termed T suppressor factor (TsF). It had a molecular weight of 50 kDa as estimated by gel filtration on Sephadex G-100 and filtration using Amicon filters, and was stable to heating at 56 °C. It could be specifically absorbed by and eluted from affinity column made by Sepharose beads with attached TNP hapten but not from column with OX-conjugated beads. The production of TsF in vitro was inhibited by treatment of Ts cells with anti-Thy-1 (now CD90) antibodies and complement. It was also shown that TNP-TsF carries I-J determinant (Zembala et al. 1975; Asherson and Zembala 1975, 1976; Claman 1976). Later Claman (1976) and Moorhead (1977a, b) repeating these experiments in different haptenic systems have demonstrated that lymphoid cells from mice injected i.v. with dinitrophenol (DNP) hapten followed by skin sensitization with DNFB inhibit the passive transfer of CS and produce in vitro soluble TsF (Phanuphak et al. 1974). The T lymphocyte origin of suppressor cells was proved by abolition of inhibitory effect by elimination of T cells from suppressive population by anti-Thy-1 antibodies (Moorhead 1977a). DNP-specific Ts factor had a molecular weight between 35 and 65 kDa. DNP-specific Ts cells blocked CS rather at its afferent (cell proliferation) than effector stage. DNP-TsF has no immunoglobulin determinants since it could not be removed on affinity columns of polyvalent anti-mouse-immunoglobulin antibodies. Further, and for its action, no identity of K or D genes of the H-2 complex between the producers of TsF and donors of treated immune cells was required (Moorhead 1977b). The authors argued that DNP-TsF is a bivalent molecule with one specificity for the H-2 complex and a second for the hapten DNP. Also in other haptenic systems (4-hydroxy-3-nitrophenyl-acetyl, NP; and azobenzenearsonate, ABA) suppressor cells and corresponding suppressor factors were described by Benacerraf’s group at Harvard (Bach et al. 1978; Sunday et al. 1981). Later Ptak et al. (1980) proposed using a uniform procedure for production of anti-hapten TsF with desired specificity. In all studied so far hapten-specific suppressor systems, it was demonstrated that Ts factors act strictly in antigen-specific manner. Thus, TsF produced by TNP-activated Ts cells suppressed TNP-immune effector cells but had no activity in the oxazolone system and, what more surprising, it also was inactive in the DNP system (and vice versa) although DNP and TNP haptens differ only by one nitro group.

In our early experiments using affinity chromatography, we confirmed the original Asherson and Zembala (1974b) data using TsF with two different Ag specificities (TNP and OX). Hapten specificity was confirmed for these factors in criss-cross tests. They occurred as proteins with molecular size ranging from 70 to 400 kDa as estimated by gel filtration. The larger weight species were probably oligomers of 68 kDa monomeric polypeptide chains although minor species corresponding to 25–30 kDa were also observed (Rosenstein et al. 1981). Suppressor activity was found only in fractions of higher molecular weight (Ptak et al. 1983b). We did not find immunoglobulin markers in the affinity purified TsF, and they also did not express antigenic determinants coded by MHC genes (Ptak and Gershon 1982). We admitted that these findings did not exclude the possibility that an additional component (MHC positive) could be added at the site where suppression is being effected (Ptak et al. 1983a).

To summarize, the results obtained hitherto by several independent groups have clearly shown that Ts cells are generated by single or multiple injections of free or cell-bound hapten, while to produce TsF mice need two separate antigen administrations, i.v. as above and epicutaneous by skin painting. In general, both Ts cells and TsF work similarly by suppressing the adoptive transfer of CS effector cells. As found by us later Ts cells are FoxP3 negative, i.e., are most likely not progeny of natural suppressor T cells (Bryniarski et al. 2013a, b).

### Two Components of TsF

The experimental data obtained so far posed two essential questions regarding the structure, the cellular target and the mechanism of action of TsF.

1. Why to initiate production of TsF two different antigen presentations (i.e., i.v. and epidermal) is necessary: is it because epidermally applied antigen serves as a boost to Ts cells actually primed by i.v. antigen administration, so TsF production is like an anamnestic response or, alternatively, is it due to generation of two different cells which require diverse modes of antigen presentation and then produce distinct molecules which only in combination have suppressor activity.
2. Is the effector cell of contact sensitivity the direct target of action of TsF or are some additional mechanisms involved. Experiments by Benacerraf’s group at Harvard and Asherson and Zembala (Bach et al. 1978; Germain and Benacerraf 1981; Zembala et al. 1982b) suggested that the Ts cells or their products enter multiple interactions with production of intermediary factors and the effector cell of such circuit produces the final suppressing molecule. Our experiments also suggested the existence of an intermediary cell and the action of intermediary factors (see below) (Flood et al. 1986).

The mechanism of anamnestic response seemed to us less likely as recurrent i.v. administrations of antigen did not lead to triggering of TsF production. The hypothesis that two independent antigen administrations (i.v. injection and contact sensitization) trigger two different cells was tested as follows. Supernatants resulting from separate cultures of lymph node and spleen cells from mice either tolerized i.v. with TNBSA (or TNP-labeled erythrocytes) or only contact sensitized with PCL were tested separately or after their co-incubation for treatment of CS effector cells in adoptive transfer. As control, supernatant of culture of cells from mice that underwent a complete sequence of tolerization procedures were used. CS reactions were inhibited only in recipients of effector cells incubated with complete supernatant or mixed fractions. It was concluded that two different cell populations were responsible for production of subfactors, which together form an active TsF (Ptak et al. 1982b).

Our experiments then showed that the two rounds of immunization (i.v. and epicutaneous) activate two cells with different Ly phenotypes. T lymphoid cells from PCL-immunized mice producing subfactor PCL-F expressed Ly1 (now CD5) phenotype while TNBSA-F was made by Ly2+ (now CD8+) T cells from mice immunized by TNP-conjugated mouse red blood cells (TNP-MRBC), but only the molecule produced by Ly1+ cells bound the antigen as confirmed using affinity columns (van Loveren et al. 1984). Similarly, two subfactors also were induced in mice analogously tolerized to oxazolone. At the time (i.e., 1982) when these experiments were performed Ly1 (CD5) phenotype was regarded as characteristic of helper T cells and for this reason we described (as turned out later erroneously) PCL-F as a T cell product. We could not confirm the postulate that these two subfactors work in combination as a single unit, since solid phase and fluid-phase conditions failed to deliver any evidence that they associate with one another (Rosenstein et al. 1981). Further attempts to closer characterize the TNBSA-F molecule were only partially satisfactory. In Askenase lab it was found that a part of its activity may be due to the presence of PGE2 prostaglandin. It was demonstrated that when Ts cells are cultured in the presence of indomethacin, a cyclooxygenase inhibitor, the produced supernatant contained less active TsF (Kato and Askenase 1984). TsF activity could be restored by addition of PGE2, followed by prolonged and repeated dialysis to remove free PGE2 (Ferreri et al. 1993). TsF is excreted in exosomes (see *infra*) that might be permeable for different molecules, as shown by us later for miRNA (Bryniarski et al. 2013b), which thus can modify their function. Askenase and co-workers (Ferreri et al. 1993) found using chromatographic separation on Sepharose G-200 and HPLC technique that TNBSA-F had molecular weight about 35-55 kDa and did not contain regulatory cytokines (like TGF-beta and IFN-gamma) and neither influenced the production of cytokines by immune cells.

### Light Chains

PCL-F on its own had interesting biological activity in vivo. We found unexpectedly that purified PCL-F injected intravenously into mice that were then challenged on ears with hapten was responsible for an early occurring, within 30 min after antigen deposition, strong inflammatory reaction manifested as ear swelling (Ptak et al. 1982a). This reaction was not MHC restricted (Ptak et al. 1991). A similar local reaction could be produced by i.v. injection of TNP-specific monoclonal IgE antibodies and it could not be demonstrated in mast cell-deficient mice, thus suggesting that in both cases reactions were dependent on mast cell release of mediators (Askenase et al. 1983). Indeed, as shown by us later, the early ear swelling 2 h after challenge of immune animals is due to degranulation and liberation of vasoactive amines by mast cells coated with antigen-specific light chains produced by B1a B cells (Szczepanik et al. 2003). These observations led us to reevaluate our former experiments which we interpreted as indicating that PCL-F was a product of T helper cells (Ptak et al. 1983b) since it had no immunoglobulin determinants. In a series of next experiments, it was shown clearly that the Ly1 (CD5) positive cell is not a T cell but is in fact a B1 lymphocyte (exactly belonging to B1a subpopulation), and PCL-F is an IgM antibody or its kappa light chain (Kerfoot et al. 2008; Paliwal et al. 2002; Tsuji et al. 2002; Itakura et al. 2005). IgM and light chains presumably use some other mechanisms to coat cells and excreted exosome vesicles, since they do not bind to FcR for IgE. Perhaps IgM and light chain coating is provided by their association with some cell and exosome surface-bound lipid molecules (Hutchinson et al. 2012). These results were in agreement with our former data showing that low molecular weight fraction of PCL-F (25 kDa) is responsible for mediation of an early 2 h CS reaction (Ptak et al. 1983b), while higher weight PCL-F molecules, presumably of polymeric structure, participate in the suppression mechanism. Interestingly, skin painting with PCL induces very rapid production of IgM antibodies (within 24 h) by B1 cells since their presence can be detected either by ear swelling reaction in immunized mice or by their presence in circulation (Itakura et al. 2005; Tsuji et al. 2002). The local effect of PCL-F (or correctly IgM antibodies) is also due to its reaction with skin-applied hapten, which leads to the local complement activation and formation of C5a peptide which in turn activates mastocytes (Tsuji et al. 2000). Activation of complement and production of vasoactive mediators by mast cells facilitate influx to the site of reaction of antigen-specific T cells that mediate the late phase of CS reaction. In effect, the late 24 h contact sensitivity reaction is initiated by B1 cell antibody-dependent mechanisms. In B1 cell-deficient xid mice, the early phase is missing and the late phase of CS is defective (Paliwal et al. 2002).

During antibody synthesis, immunoglobulin-free light chains (FLC) are secreted as monomers or homodimers in excess over heavy chains by plasma cells. For a long time, they were regarded as devoid of any biological function. But recently biological activity of FLC was recognized in various immune phenomena (Redegeld and Nijkamp 2003). It has been shown that they can mediate type I hypersensitivity reactions and are involved in initiation of an early phase of CS (Groot Kormelink et al. 2012). FLC can also associate with certain lipids present in cell membranes (Hutchinson et al. 2012). Our former and present experiments demonstrate that the same antigen-binding molecule (IgM or its light chain) may participate in two opposite immune reactions, initiating and facilitating contact sensitivity reactions or depressing it by forming a part of suppressor molecule warranting in both cases their antigen specificity. These tests explain why in our former experiments we were not able to find immunoglobulin determinants on PCL-F produced by B1 cells. Classical polyvalent anti-immunoglobulin antibodies bind poorly to IgM and light chains released by B1 cells (Askenase et al. 2005). Our misinterpretation of the character of PCL-F producing cell as T lymphocyte is due to fact that B1a cells are weakly CD5+ and CD90+ regarded previously as characteristic for T cells. B1 cells also have conventional B cell markers like CD19, CD22, B220 and CD43 unknown at the time of our work (Szczepanik et al. 2003).

The presence of common MHC determinants on TsF is controversial. In our experiments, they were absent. Many authors have found that suppressor molecules, independently of their antigen specificity, are I-J positive (Greene et al. 1977; Sy et al. 1980; Ptak et al. 1983a). Character and function of this molecule, which for some time was regarded as a product of I region of mouse MHC, is unknown although a series of frequently mutually excluding explanations were proposed. The existence of I-J molecule (Volpe 1993) and its possible involvement in TsF activity remains unclear. For this reason, we omit in the present summary this problem since it does not change the central part of our considerations. However, since TsF (i.e., miRNA and IgM-derived-free light chains) associated with Ts cell-derived suppressive exosomes (see *infra*), it is possible that I-J (but also other MHC determinants) may rather be a part of exosome cellular envelope and not a part of TsF itself.

### Third Cell

Since the passive transfer of CS can be prevented by incubation of immune cells with TsF this may suggest that the target of TsF is the effector T cell of the CS reaction. However, adoptively transferred cells usually are a mixture of T and B lymphocytes, macrophages and other cells. Thus, the suppressive effect of TsF could be mediated indirectly by other cell types. Zembala and Asherson (1974; Asherson and Zembala 1974b) found that TsF can be adsorbed by peritoneal macrophages and then these cells, when added to passively transferred immune cells, inhibit in recipients the CS reaction. This suggested that macrophages may be the primary target cell for TsF action. In subsequent experiments, Ptak et al. (1978a) found that macrophages incubated with TsF and then exposed to corresponding antigen produce a low molecular weight (approximately 10 kDa) molecule called thereafter MSF (macrophage suppressor factor), which is responsible for suppression and, in contrast to TsF, has no antigen specificity, i.e., equally well inhibits the transfer by cells immune to homologous or heterologous antigen. TsF seemed to bind to macrophages via cell surface structures (like Fc receptors) that could be blocked with heat-aggregated gamma globulins (Ptak et al. 1977). Macrophage membranes by absorption of lymphocyte products can interfere with transmission of cell messages (Gershon et al. 1976; Ptak et al. 1978a, b). Also heat-killed macrophages bind TsF but as expected fail to produce MSF (Gershon et al. 1976). MSF is apparently not identical with TsF because of its low molecular weight (10 versus 60 kDa) and antigen non-specific action. Inhibitors of protein synthesis (cycloheximide and emetine) and RNA synthesis (actinomycin D) completely block MSF synthesis by macrophages, while blocking production of prostaglandins has no influence (Marcinkiewicz and Ptak 1980). Interesting is also the selective inhibition by MSF of CD4+ T cells mediating CS reaction and lack of influence on CD8+ suppressor T cells. The action of MSF is transitory, i.e., when adoptively transferred recipients were tested immediately after transfer of MSF-treated immune cells the reaction was inhibited, but postponing the test for several days showed no inhibition; indicating that the effector cells recover and that MSF is not cytotoxic (Ptak et al. 1978a). Moreover, hapten-labeled macrophages treated with TsF lost their antigen-presenting capability and when injected into recipients were unable to induce CS effector cells. Ptak and Gershon (1982) termed this phenomenon “Immunological agnosis”.

Other experimental data indicate the existence of an alternative mechanism of suppression by TsF. Zembala et al. (1982b) showed that immune cells from mice pretreated with cyclophosphamide are unaffected by incubation in TsF. The cell responsible for suppressor mechanisms removed by cyclophosphamide treatment is a CD8+, I-J+, Fc+ T cell called by the authors a “T acceptor cell”. It produces an antigen non-specific factor with molecular weight of 30–50 kDa. Later Flood et al. (1986) confirmed the action of cyclophosphamide and additionally found that the responsible CD8+ cell adheres to *Vicia villosa* lectin. In extension of such studies, it was speculated that various cellular interactions and production of several intermediary factors are necessary to produce suppression. Since regulatory pathways encompassing as postulated three types of Ts cells are complicated, and as suggested include antigen- and idiotype-specific interactions, and experiments were mainly done in laboratories of Benacerraf (Bach et al. 1978; Benacerraf 1978; Germain and Benacerraf 1981; Greene and Benacerraf 1980; Jayaraman and Bellone 1986; Sunday et al. 1981) and Asherson (Zembala et al. 1982b), we omit further deliberation on this topic in our present discussion.

### New Age of TsF Research: Discovery of Treg Cells

The idea of Ts cells dominating in 1970s led to description in a wide range of systems of various inhibitory factors differing in specificity and mode of action (Taussig 1980; Webb et al. 1994). A decade later their existence has been questioned (Arnon and Teitelbaum 1993; Bloom et al. 1992) or even negated (Möller 1988) because of lack of specific cell surface markers on Ts cells, futile attempt to produce their clones and the indeterminate molecular basis of the soluble suppressor factors produced by these cells. In effect, studies on TsF were heavily curtailed for several years or abandoned, and available financial support was greatly reduced. Today, because of discovery of natural suppressor cells by Sakaguchi in 1995 (Sakaguchi 2000), that control autoreactive T cells in vivo in an antigen non-specific manner and finding of their specific markers (e.g. FoxP3), there is an important resumption of interest in suppression mechanisms, with a significant nomenclature change, i.e., the term suppressor cells was changed, and now exists as T regulatory cells. Unfortunately, this is a rather ambiguous term since it does not precisely indicate whether down or up-regulatory function of cells is concerned.

### Discovery that miRNA is a Part of TsF

At present substantial attention is focused on the regulation of biological function of various cell populations, including cells of the immune system, by the action of small interfering RNA (siRNA) molecules like miRNAs. siRNA may regulate the immune response (Xiao and Rajewsky 2009) by influence on differentiation of immune cells (Tsitsiou and Lindsay 2009). Our recent experiments proposed for a first time a novel siRNA-dependent mechanism of suppressor cell action.

Bryniarski and Ptak in collaboration with Askenase (Yale University) using molecular methodology proposed a new model of the nature and origin of hapten-specific TsF (Bryniarski et al. 2013b). They differentially treated suppressive Ts cell culture supernatant containing TsF with RNase and tested the resulting product for its biological activity. Such treatment removed suppressive activity of TsF, which suggested the participation of RNA in TsF-mediated immune regulation. To test this hypothesis, DNA/RNA material of Ts cell culture supernatant was subjected to phenol–chloroform extraction (PCE) according to the Chomczynski and Sacchi (1987) method and tested in adoptive transfer of CS effector cells. Interestingly, the DNA/RNA extract from TsF-containing supernatant suppressed adoptively transferred CS reactions and this effect also was inhibited by pretreatment of the PCE extract with RNase, while pretreatment with DNase had no effect. Further purification of suppressive DNA/RNA extract on Qiagen chromatographical columns resulted in separated fractions of DNA and RNA, from which only purified RNA material suppressed adoptively transferred CS responses. In addition, treatment of the isolated TsF-derived RNA with RNase A and with RNase III specific for double stranded RNA (dsRNA) also blocked the suppressive activity of the assayed fractions (Sikora et al. 2012). This led to the assumption that dsRNA, especially miRNA, could be responsible for the observed regulatory effect of TsF actions. Isolated suppressive RNA material was then electrophoretically separated by sizing on 12 % polyacrylamide gel and compared to RNA size markers. Separated RNA from resulting bands was singly eluted from gel and tested for biological activity, which was demonstrated only for small RNA in the range of 75 base pairs, confirming the involvement of small dsRNA molecules in the mechanism of TsF-mediated immune suppression. In parallel the question arose, how dsRNA molecules present in suppressive supernatant and in blood plasma of tolerized mice can be functionally active in this environment known to be rich in RNases; and exosomes contain inside or on their membranes a variable spectrum of molecules, including proteins, lipids, DNA and RNA, that they can deliver to acceptor cells. Thus, different assays to detect various possible protective entities, such as protein chaperones and exosomes, were employed. Nanoparticle tracking analysis (NTA) and electron microscopy followed by cytofluorimetric analysis allowed the demonstration of exosomes in ultracentrifuged (100,000*g*) pellet from TsF. The previously reported capacity of TsF to bind antigen enabled us to perform antigen affinity column chromatography to successfully separate the subpopulation of exosomes (12 % of the total) that were able to suppress adoptive transfer of CS effector cells. Antigen affinity chromatography procedure to separate exosomes was used experimentally for the first time and allowed us to prepare pure exosome probes for molecular and bioinformatic analysis. In cooperation with the Tuschl laboratory at The Rockefeller University, RNA isolated from chromatographically separated antigen-binding exosomes was cloned and sequenced, which defined the possible microRNA (miRNA) molecules as candidates responsible for TsF exosome activity. To test the exact miRNA mediating the suppression, we first used miRNA antagonists (anti-miRs) to the most frequent miRNAs shown to be present in assayed suppressive exosomes. This showed that only anti-miR to miRNA-150 reversed the suppressive activity of exosomes in the adoptive transfer of CS effector cells. Further, tolerization of miRNA-150^−/−^ (double knockout) mice (obtained by courtesy of Klaus Rajewsky, Harvard University) by i.v. injection of OX-labeled erythrocytes completely failed to inhibit active OX-induced CS reactions in these animals. Finally, we showed that Ts cells from tolerized miRNA-150^−/−^ mice, when cultured, release functionally non-active exosomes. Very significantly, incubation of these non-active exosomes with synthetic miRNA-150 (and not various controls) restored their suppressive activity, proving that miRNA-150 molecules mediate TsF suppressor function (Bryniarski et al. 2013b). Since IL-2 plays a role in DTH reactions, Askenase also investigated the effect of TsF-derived exosomes that occurred to be inhibitory (Bryniarski et al. 2013b), on the responsiveness of targeted HT-2 cell line to IL-2 in an in vitro assay (Ferreri et al. 1993).

In parallel, we have tried to define the factor responsible for antigen specificity of exosome suppressive activity, which also was confirmed by us in criss-cross experiments and specific binding to antigen in affinity chromatographic columns. Noteworthy, attempts to use antigen affinity chromatography to fractionate DNA/RNA extracted with phenol–chloroform from TsF-containing supernatant were unsuccessful, which suggested that the antigen specificity capacity of TsF is mediated by protein entity removed during the PCE procedure that also destroyed exosomes. Considering previous reports, we cytofluorymetrically analysed exosomes for the potential presence of immunoglobulins on their surface. This analysis demonstrated the surface expression of antibody kappa light chains and failed to show heavy chains or complete immunoglobulin on the exosomes. Therefore, we concluded that antibody light chains could be responsible for antigen specificity of TsF. To confirm this hypothesis, we then tolerized immunoglobulin-deficient JH^−/−^ mice to generate TsF. The resulting exosomes did not suppress adoptively transferred CS effector cell responses, but were able to antigen non-specifically inhibit the responsiveness of HT-2 cell line to IL-2 (see *supra*) in an equal manner to TsF suppressive exosomes from wild type mice. This allowed us to conclude that the observed lack of suppressor activity of exosomes from JH^−/−^ mice was due to the absence of an immunoglobulin component and not miRNA-150 nor other subfactor. To confirm this assumption, exosomes isolated from hapten tolerized JH^−/−^ mice that were non-suppressive, were supplemented with TNP-specific monoclonal antibody either light or heavy chains and then incubated with TNP-immune CS effector cells, that were then adoptively transferred into naive recipients. Addition of TNP-specific antibody light chains enabled JH^−/−^ mouse exosomes to suppress CS responses, while supplemented heavy chains were inactive (Bryniarski et al. 2013b). This observation proved that antigen specificity of TsF is mediated by the hapten-specific antibody light chains produced by B1 lymphocytes and present on the surface of regulatory exosomes containing miRNA-150. According to previous research of the Askenase group (Kerfoot et al. 2008; Itakura et al. 2005; Paliwal et al. 2002; Tsuji et al. 2002), B1 cells are activated by hapten contact immunization and release specific IgM and antibody light chains into body fluids that are responsible for an early component of elicited CS reaction. As mentioned above, PCL-F subfactor of TsF expresses analogous biological activity, and now it is shown that B1 cell-derived antibody light chains can coat exosomes to render them antigen specifically suppressive; suggesting the identity of these components.

At the same time, it remained unexplained how extracted suppressive RNA could be functionally active without the protection of exosomes. Since assayed CS effector cells treated with suppressive RNA are in fact a mixture of various cell populations, including B1 lymphocytes, we tested the possibility that the assayed cells also release exosomes that may potentially bind or engulf RNA molecules. This assumption seemed to be strongly supported by the previous finding that miRNA-150 in exosomes can be blocked by anti-miRs, which suggested the ability of exosomes to engulf or associate with small nucleic acid molecules. To test this hypothesis, we have isolated B1 lymphocytes from contact hapten-immunized donor spleens and lymph nodes and cultured them in the same conditions as are Ts lymphocytes. The resulting supernatant was processed as for exosome harvest and finally pelleted by ultracentrifugation of putative B1 cell-derived exosomes that then were incubated with either isolated suppressive mixed RNA derived from TsF or synthetic miRNA-150 alone. After washing by ultracentrifugation at 100,000*g* to remove free RNA, the pelleted RNA-pulsed B1 cell exosomes were incubated with CS effector cells that, after washing to remove the exosomes, were then adoptively transferred into naive mice. In effect, treatment of CS effector cells with B1 cell-derived exosomes pre-incubated with both, isolated RNA and miRNA-150, resulted in significant suppression of transferred CS response, while B1 cell exosomes not treated with the RNA were inactive. This observation allowed us to consider that isolated (extracellular) miRNA, likely protected from RNases by a chaperone Argonaute protein, can circulate and act eventually in an endocrine manner to suppress the effector T cell mixture by entering to or associating with antigen-specific exosomes released by the targeted cell population, in this case B1 cell companions of the effector T cells. We named this phenomenon “passive transfection” of exosomes with miRNA (Bryniarski et al. 2014b). Indeed, our preliminary results suggest that extracellular miRNA could be protected by attachment to protein chaperones of Argonaute family and that miRNA transfection of exosomes could be SID-1 dependent (Bryniarski et al. 2014b). However, the exact mechanism of this phenomenon remains the subject of our ongoing studies.

Explanation of the nature of TsF as exosomes carrying suppressive miRNA-150 and coated with antigen-specific antibody light chains allowed for further studies of their immune activity. Assayed exosomes isolated from Ts cell culture supernatant were shown to be able to dose dependently and antigen specifically inhibit both active (after intraperitoneal injection before either contact immunization or challenge) and adoptively transferred CS reaction (when incubated with CS effector cells) as well as to suppress the clinical symptoms of active allergic response (after intraperitoneal injection 24 h after elicitation of CS) (Bryniarski et al. 2014a). Interestingly, the alleviation of allergic symptoms was observed in mice even when separated TsF exosomes were administered orally (Bryniarski et al. 2013b). Noteworthy, exosomes separated from blood plasma of tolerized mice express similar suppressive activity. Additionally, we have demonstrated that TsF exosomes are able to inhibit in antigen non-specific manner HT-2 cell line responsiveness to IL-2, which is consistent with reports concerning miRNA-150 involvement in regulation of hematopoiesis. Thus, our data strongly suggest the participation of TsF exosomes in suppression of various biological processes, apart from immunosuppressive function.

To summarize, during our ongoing studies in murine TNP- and OX-induced tolerance model, we have confirmed previous observation of Ptak et al. (1982b) that TsF is not a single entity but in fact consists of the products of two different cell subsets (Bryniarski et al. 2012, 2013a, b), defined as T CD8+ suppressor lymphocytes (Ly2+ cells), source of the miRNA-150 containing exosomes, and B1 CD5+ cells (Ly1+ cells, previously incorrectly identified as T helper lymphocytes), source of antibody-free light chains coating suppressive exosomes for antigen specificity. Intravenous administration of hapten-labeled syngeneic erythrocytes induces the release of miRNA-150 in exosomes (previously termed TNBSA-F) by Ts CD8+ cells, whereas their hapten specificity is ensured by the surface coat with antibody light chains (previously termed PCL-F) secreted by B1 lymphocytes activated by contact immunization with the same hapten (Bryniarski et al. 2012, 2013a, b). This is consistent with previous observation that TsF acts as mixture of TNBSA-F and PCL-F (Ptak et al. 1982b). Interestingly, T CD8+ suppressor lymphocytes do not express FoxP3 marker and differ from classical T FoxP3+ regulatory cells (Bryniarski et al. 2013a, b), which was proved by successful tolerization of DEREG mice depleted of FoxP3-expressing cells. Noteworthy, previous results suggest that PCL-F as an oligomer positively mediates early 2 h CS reaction after elicitation, while as polymer it acts as subfactor of TsF (Ptak et al. 1983b). Our preliminary data imply that B1 lymphocytes, apart from light chains, are also able to release antigen-specific exosomes that could be transfected with miRNA (Bryniarski et al. 2014b). Thus, in the view of our studies, this could be interpreted as follows. Light chains as PCL-F monomers initiate CS response, which was recently confirmed (Paliwal et al. 2002) as well as coat T cell-derived suppressor exosomes, while putative B1 cell-derived exosomes characterized as PCL-F polymers may suppress CS reaction. To our best knowledge, this is the first information in the literature about the antigen-specific action of miRNA in down-modulation of immune response.

### Postulated Mechanism of Suppressive Exosome Action

Our former experiments suggested that macrophages may be the target cell population for TsF suppressive action. Current preliminary results seem to confirm this suggestion (Nazimek et al. 2012, 2013). It was demonstrated that treatment of macrophages with T CD8+ suppressor cell-derived TNP-specific exosomes carrying miRNA-150 impairs their ability to induce cellular and humoral immune responses in vivo and affects in vitro their phagocytic activity as well (Nazimek et al. 2013, 2014a, b). Nevertheless, the mechanism of exosomal miRNA-150 regulatory action is still an open question and remains our main research interest. However, TsF subfactors (i.e. IgM, antibody light chains and miRNA) are most likely released by responsible cells both in exosome-bound form and also simultaneously as free entities. Thus, doubly equipped TsF exosomes are able to reach the desirable antigen-bearing cell (macrophages and/or T cell subset) owing to a coat of antigen-specific antibody light chains to deliver selected miRNA, which then inhibits proinflammatory signaling pathways via RNA-interference mechanisms in targeted cells.

## Conclusions

After several years of extensive criticism of TsF existence, our discovery allowed to characterize its nature as regulatory miRNA-150 carried by antigen-specific exosomes that are able to suppress active and adoptively transferred CS reaction in mice. Our experiments show that function of exosomes can be deliberately manipulated by incubation with regulatory RNA of choice thus changing their RNA-dependent function or alternatively by addition of immunoglobulin-free light chains of required antigen specificity to achieve the desirable targeting into recipient cells. These observations seem to have potential to be considered in therapeutic use in human diseases associated with immune regulation disorders. Furthermore, Karlsson et al. (2001; Ostman et al. 2005) found in sera of ovalbumin (OVA)-fed animals MHC class II positive OVA-specific cellular vesicles produced by intestinal epithelial cells. These vesicles could suppress OVA-specific immune response by presenting antigen in tolerogenic form and authors proposed to name them tolerosomes. Even though the vesicles described by Karlsson are structures presenting antigen in tolerogenic form and our exosomes carry an RNA suppressive moiety the term “tolerosomes” applies to both situations, since it is adequate to both their morphology (tolero***somes***) and function (***tolero***somes). Thus, we would recommend this name to be included into the immunological lexicon and used to describe all extracellular vesicles, independently of their origin and mechanism of suppression of immune responses.

## References

[CR1] Arnon R, Teitelbaum D (1993). On the existence of suppressor cells. Int Arch Allergy Immunol.

[CR2] Asherson GL, Ptak W (1968). Contact and delayed hypersensitivity in the mouse. I active sensitization and passive transfer. Immunology.

[CR3] Asherson GL, Ptak W (1970). Contact and delayed hypersensitivity in the mouse. III. Depression of contact sensitivity by pre-treatment with antigen and the restoration of immune competence in tolerant mice by normal lymphoid and bone marrow cells. Immunology.

[CR4] Asherson GL, Zembala M (1974). Suppression of contact sensitivity by T cells in the mouse. I. demonstration that suppressor cells act on the effector stage of contact sensitivity; and their induction following in vitro exposure to antigen. Proc R Soc Lond.

[CR5] Asherson GL, Zembala M (1974). T cell suppression of contact sensitivity in the mouse. III. The role of macrophages and the specific triggering of nonspecific suppression. Eur J Immunol.

[CR6] Asherson GL, Zembala M (1975). Inhibitory T cells. Curr Top Microbiol Immunol.

[CR7] Asherson GL, Zembala M (1976). Suppressor T cells in cell-mediated immunity. Brit Med Bull.

[CR8] Askenase PW, Middleton E (2014). Effector and regulatory mechanisms in delayed-type hypersensitivity. Allergy—principles and practise.

[CR9] Askenase PW, Rosenstein RW, Ptak W (1983). T cells produce an antigen-binding factor with in vivo activity analogous to IgE antibody. J Exp Med.

[CR10] Askenase PW, Itakura A, Leite-de-Moraes MC, Lisbonne M, Roongapinun S, Goldstein DR, Szczepanik M (2005). TLR-dependent IL-4 production by invariant Valpha14+ Jalpha18+ NKT cells to initiate contact sensitivity in vivo. J Immunol.

[CR11] Bach BA, Sherman L, Benacerraf B, Greene M (1978). Mechanism of regulation of cell-mediated immunity. II. induction and suppression of delayed-type hypersensitivity to azobenzenearsonate-coupled syngeneic cells. J Immunol.

[CR12] Battisto JR, Bloom BR (1966). Dual immunological unresponsiveness induced by cell membrane coupled hapten or antigen. Nature.

[CR13] Battisto JR, Bloom BR (1966). Mechanism of immunologic unresponsiveness: a new approach. Fed Proc.

[CR14] Battisto JR, Chase MW (1955). “Immunological paralysis” in guinea pigs fed chemical allergens. Fed Proc.

[CR15] Benacerraf B (1978). Suppressor T cells and suppressor factor. Hosp Pract.

[CR16] Bloom BR, Salgame P, Diamond B (1992). Revisiting and revising suppressor T cells. Immunol Today.

[CR17] Bryniarski K, Nazimek K, Sikora E, Nowak B, Ptak M, Askenase PW, Ptak W (2012). miRNA-150 inhibits contact sensitivity response via antigen-specific exosomes produced by T suppressor cells. Immunology.

[CR18] Bryniarski K, Nazimek K, Sikora E, Ptak M, Askenase PW, Ptak W (2013). TCD8+ suppressor cells produce antigen-specific exosomes carrying miRNA-150 to inhibit contact sensitivity response. Front Immunol.

[CR19] Bryniarski K, Ptak W, Jayakumar A, Püllmann K, Caplan M, Chairoungdua A, Lu J, Adams B, Sikora E, Nazimek K, Marquez S, Kleinstein SH, Sangwung P, Iwakiri Y, Delgato E, Redegeld F, Blokhuis BR, Wojcikowski J, Daniel AW, Groot TK, Askenase PW (2013). Antigen specific, antibody coated, exosome-like nanovesicles deliver suppressor T cell miRNA-150 to effector T cells in contact sensitivity. J Allergy Clin Immunol.

[CR20] Bryniarski K, Nazimek K, Martin E, Ptak M, Askenase PW, Ptak W (2014). T CD8 + cell-derived exosomal miRNA suppresses induction and effector phases of murine contact sensitivity as well as symptoms of active allergy. Centr Eur J Immunol.

[CR21] Bryniarski K, Ptak W, Nazimek K, Askenase PW (2014). Passive transfection of antigen-specific exosomes with immunoregulatory miRNA-150 to suppress contact sensitivity. Centr Eur J Immunol.

[CR22] Chase MW (1945). The cellular transfer of cutaneous hypersensitivity to tuberculin. Proc Soc Exp Biol Med.

[CR23] Chase MW (1946). Inhibition of experimental drug allergy by prior feeding of the sensitizing agent. Proc Soc Exp Biol NY.

[CR24] Chase MW (1967). Hypersensitivity to simple chemicals. Harvey Lect.

[CR25] Chase MW, Maguire HC (1973). Studies on the sensitization of animals with simple chemical compounds. XIV. Further studies on sensitization of guinea pigs with picric acid. Int Arch Allergy.

[CR26] Chomczynski P, Sacchi N (1987). Single step method of RNA isolation by acid guanidinium thiocyanate-phenol-chloroform extraction. Anal Biochem.

[CR27] Claman HN (1976). Tolerance and contact sensitivity to DNFB in mice. V. Induction of tolerance with DNP compounds and with free and membrane associated DNFB. J Immunol.

[CR28] Crowle AJ (1960). Tuberculin skin reactions in mice hypersensitized by vaccination with living avirulent tubercle bacilli. Amer Rev Resp Dis.

[CR29] Crowle AJ, Crowle CM (1961). Contact sensitivity in mice. J Allergy.

[CR30] Ferreri NR, Herzog WR, Askenase PW (1993). Inhibition of IL-2 dependent proliferation by a prostaglandin-dependent suppressor factor. J Immunol.

[CR31] Fidler JM, Golub ES (1973). Immunological tolerance to a hapten. I. Induction and maintenance of tolerance to trinitrophenyl with trinitrobenzene sulfonic acid. J Exp Med.

[CR32] Flood P, Ptak W, Green DR (1986). Mechanism of action of a T suppressor factor (TsF) in contact sensitivity: the T cell target for TsF activity in adoptive transfer of immunity is not the effector cell. J Immunol.

[CR33] Germain RN, Benacerraf B (1981). A single major pathway of T lymphocyte interactions in antigen-specific immune suppression. Scand J Immunol.

[CR34] Gershon RK, Kondo K (1970). Cell interactions in the induction of tolerance: the role of thymic lymphocytes. Immunology.

[CR35] Gershon RK, Kondo K (1971). Infectious immunological tolerance. Immunology.

[CR36] Gershon RK, Cohen P, Hencin R, Liebhaber SA (1972). Suppressor T cells. J Immunol.

[CR37] Gershon RK, Askenase PW, Gershon MD (1975). Requirement for vasoactive amines for production of delayed-type hypersensitivity skin reactions. J Exp Med.

[CR38] Gershon RK, Eardley DD, Ptak W (1976). Functional inactivation of suppressor T cells by heat-killed macrophages. Nature.

[CR39] Green DR, Ptak W (1986). Contrasuppression in the mouse. Immunol Today.

[CR40] Greene MI, Benacerraf B (1980). Studies on hapten specific T cell immunity and suppression. Immunological Rev.

[CR41] Greene MI, Pierres A, Dorf ME, Benacerraf B (1977). The I-J subregion codes for determinants on suppressor factor(s) which limit the contact sensitivity response to picryl chloride. J Exp Med.

[CR42] Groot Kormelink T, Askenase PW, Redegeld FA (2012). Immunobiology of antigen-specific immunoglobulin free light chains in chronic inflammatory diseases. Curr Pharm Des.

[CR43] Hutchinson AT, Jones DR, Raison RL (2012). The ability to interact with cell membranes suggests possible biological roles for free light chain. Immunol Lett.

[CR44] Itakura A, Szczepanik M, Campos RA, Paliwal V, Majewska M, Matsuda H, Takatsu K, Askenase PW (2005). An hour after immunization peritoneal B-1 cells are activated to migrate to lymphoid organs where within 1 day they produce IgM antibodies that initiate elicitation of contact sensitivity. J Immunol.

[CR45] Jayaraman S, Bellone CJ (1986). Idiotype-specific T suppressor factor alternatively interacts with a nonspecific T-acceptor-like cell to mediate immune suppression. Cell Immunol.

[CR46] Kaplan DH, Igyarto BZ, Gaspari AA (2012). Early events in the induction of allergic contact dermatitis. Nat Rev Immunol.

[CR47] Karlsson M, Lundin S, Dahlgren U, Kahu H, Pettersson I, Telemo E (2001). “Tolerosomes” are produced by intestinal epithelial cells. Eur J Immunol.

[CR48] Kato K, Askenase PW (1984). Reconstitution of an inactive antigen-specific T cell suppressor factor by incubation of the factor with prostaglandins. J Immunol.

[CR49] Kerfoot SM, Szczepanik M, Tung JW, Askenase PW (2008). Identification of initiator B cells, a novel subset of activation-induced deaminase-dependent B-1-like cells that mediate initiation of contact sensitivity. J Immunol.

[CR50] Kops SK, van Loveren H, Rosenstein RW, Ptak W, Askenase PW (1984). Mast cell activation and vascular alterations in immediate hypersensitivity-like reactions induced by a T cell-derived antigen-binding factor. Lab Invest.

[CR51] Landsteiner K, Chase MW (1942). Experiments on transfer of cutaneous sensitivity to simple chemical compounds. Proc Soc Exp Biol Med.

[CR52] Marcinkiewicz J, Ptak W (1980). Macrophage suppressor factor in contact sensitivity. Mechanism of its release and action. Immunology.

[CR53] Möller G (1988). Do suppressor T cells exists. J Immunol.

[CR54] Moorhead JW (1977). Soluble factors in tolerance and contact sensitivity to 2,4-dinitrofluorobenzene in mice. J Immunol.

[CR55] Moorhead JW (1977). Soluble factors in tolerance and contact sensitivity to DNFB in mice. II. Genetic requirements for suppression of contact sensitivity by soluble suppressor factor. J Immunol.

[CR56] Nazimek K, Nowak B, Ptak W, Bryniarski K (2012). Exosomal T cell suppressor factor inhibits the generation of reactive oxygen intermediates in murine peritoneal macrophages. Immunology.

[CR57] Nazimek K, Ptak M, Ptak W, Bryniarski K (2013). T CD8+ suppressor cell-derived exosomes carrying miRNA-150 impair macrophage ability to induce humoral immune response in mice. Front Immunol.

[CR58] Nazimek K, Nowak B, Marcinkiewicz J, Ptak M, Ptak W, Bryniarski K (2014). Enhanced generation of reactive oxygen intermediates by suppressor T cell-derived exosome-treated macrophages. Folia Med Cracov.

[CR59] Nazimek K, Nowak B, Ptak M, Ptak W, Bryniarski K (2014). Essential role of macrophages in antigen-specific suppression of immune response mediated by T CD8 + lymphocyte-derived regulatory exosomes. Centr Eur J Immunol.

[CR60] Ostman S, Taube M, Telemo E (2005). Tolerosome-induced oral tolerance is MHC dependent. Immunology.

[CR61] Paliwal V, Tsuji RF, Szczepanik M, Kawikova I, Campos RA, Kneilling M, Rocken M, Schuurman J, Redegeld F, Nijkamp FP, Askenase PW (2002). Subunits of IgM reconstitute defective contact sensitivity in B-1 cell-deficient xid mice: *kappa* light chains recruit T cells independent of complement. J Immunol.

[CR62] Phanuphak P, Moorhead JW, Claman HN (1974). Tolerance and contact sensitivity to DNFB in mice. III. transfer of tolerance with “suppressor T cells”. J Immunol.

[CR63] Pomeranz JR (1970). Immunologic unresponsiveness following a single feeding of picryl chloride. J Immunol.

[CR64] Ptak W, Asherson GL (1969). Contact and delayed hypersensitivity In the Mouse. II. The role of different cell populations. Immunology.

[CR65] Ptak W, Gershon RK (1982). Immunological agnosis: a state that derives from T suppressor cell inhibition of antigen-presenting cells. Proc Natl Acad Sci USA.

[CR66] Ptak W, Jaszcz W (1969). Histological manifestations of the contact sensitivity to oxazolone in the mouse. I. The general characteristics of the response. Folia Histochem Cytochem.

[CR67] Ptak W, Rozycka D (1977). Split unresponsiveness to the trinitrophenyl determinant. I. Manoeuvers which suppress either humoral or cell-mediated immune responses. Eur J Immunol.

[CR68] Ptak W, Naidorf KF, Gershon RK (1977). Interference with the transmission of T-cell derived messages by macrophage membranes. J Immunol.

[CR69] Ptak W, Zembala M, Gershon RK (1978). Intermediary role of macrophages in the passage of suppressor signals between T cell subsets. J Exp Med.

[CR70] Ptak W, Zembala M, Hanczakowska-Rewicka M, Asherson GL (1978). Non-specific macrophage suppressor factor: its role in the inhibition of contact sensitivity to picryl chloride by specific T suppressor factor. Eur J Immunol.

[CR71] Ptak W, Rozycka D, Rewicka M (1980). Induction of suppressor cells and cells producing antigen-specific suppressor factors by haptens bound to self carriers. Immunobiology.

[CR72] Ptak W, Askenase PW, Rosenstein RW, Gershon RK (1982). Transfer of an antigen-specific immediate hypersensitivity-like reaction with an antigen-binding factor produced by T cells. Proc Natl Acad Sci USA.

[CR73] Ptak W, Ptak M, Rosenstein RW, Gershon RK (1982). Interactions between molecules (subfactors) released by different T cell sets that yield a complete factor with biological (suppressive) activity. Proc Natl Acad Sci USA.

[CR74] Ptak W, Gershon RK, Flood PM (1983). Mechanisms of suppression in the transfer of contact sensitivity. Analysis of an I-J+ molecule required for Ly2 suppressor cell activity. J Exp Med.

[CR75] Ptak W, Gershon RK, Rosenstein RW, Murray JH, Cone RE (1983). Purification and characterization of TNP-specific immunoregulatory molecules produced by T cells sensitized by picryl chloride (PC1F). J Immunol.

[CR76] Ptak W, Herzog WR, Askenase PW (1991). Delayed-type hypersensitivity initiation by early-acting cells that are antigen-mismatched or MHC incompatible with late-acting, delayed-type hypersensitivity effector T cells. J Immunol.

[CR77] Ptak W, Majewska M, Bryniarski K, Ptak M, Lobo MF, Zajac K, Askenase PW (2009). Epicutaneous immunization with protein antigen in the presence of TLR4 ligand induces TCR alpha-beta+ CD4+ contrasuppressor cells that reverse skin-induced suppression of Th1-mediated contact sensitivity. J Immunol.

[CR78] Redegeld FA, Nijkamp FP (2003). Immunoglobulin free light chains and mast cells: pivotal role in T-cell-mediated immune reactions?. Trends Immunol.

[CR79] Rosenstein RW, Murray JH, Cone RE, Ptak W, Iverson GM, Gershon RK (1981). Isolation and partial characterization of an antigen-specific T-cell factor associated with the suppression of delayed type hypersensitivity. Proc Natl Acad Sci USA.

[CR80] Roupe G, Ridell B (1979). The cellular infiltrate in contact hypersensitivity to picryl chloride in the mouse. Acta Derm Venereol.

[CR81] Rozycka D, Ptak W (1978). Split unresponsiveness to trinitrophenyl (TNP) determinant. Suppression of anti-TNP antibody responses by sensitization with picryl chloride. Immunology.

[CR82] Sakaguchi S (2000). Regulatory T cells: key controllers of immunologic self-tolerance. Cell.

[CR83] Sikora E, Nowak B, Nazimek K, Ptak M, Askenase PW, Ptak W, Bryniarski K (2012). Structure and function of oxazolone specific T suppressor factor in contact sensitivity response. Immunology.

[CR84] Sulzberger MB (1929). Hypersensitivity to arsphenamine in guinea-pigs. I. Experiments in prevention and desensitization. Arch Dermatol Syphilol.

[CR85] Sunday ME, Benacerraf B, Dorf ME (1981). Hapten-specific T cell responses to 4-hydroxy-3-nitrophenyl acetyl (NP). VIII. suppressor cell pathways in cutaneous sensitivity response. J Exp Med.

[CR86] Sy MS, Dietz MH, Germain RN, Benacerraf B, Greene M (1980). Antigen and receptor-driven regulatory mechanisms. IV. idiotype-bearing I-J+ suppressor T cell factors induce second order suppressor T cells which express anti-idiotypic receptors. J Exp Med.

[CR87] Szczepanik M, Akahira-Azuma M, Bryniarski K, Tsuji RF, Kawikova I, Ptak W, Kiener C, Campos RA, Askenase PW (2003). B1 B cells mediate required early T cell recruitment to elicit protein-induced delayed-type hypersensitivity. J Immunol.

[CR88] Taussig MJ (1980). Antigen-specific T-cell factors. Immunology.

[CR89] Tsitsiou E, Lindsay MA (2009). microRNAs and the immune response. Curr Opin Pharmacol.

[CR90] Tsuji RF, Kawikova I, Ramabhadran R, Akahira-Azuma M, Taub D, Hugli TE, Gerard C, Askenase PW (2000). Early local generation of C5a initiates the elicitation of contact sensitivity by leading to early T cell recruitment. J Immunol.

[CR91] Tsuji RF, Szczepanik M, Kawikova I, Paliwal V, Campos RA, Itakura A, Akahira-Azuma M, Baumgarth N, Herzenberg LA, Askenase PW (2002). B cell-dependent T cell responses: igM antibodies are required to elicit contact sensitivity. J Exp Med.

[CR92] Van Loveren H, Meade R, Askenase PW (1983). An early component of delayed-type hypersensitivity mediated by T cells and mast cells. J Exp Med.

[CR93] Van Loveren H, Kato K, Meade R, Green DR, Horowitz M, Ptak W, Askenase PW (1984). Characterization of two different Ly+ T cell populations that mediate delayed-type hypersensitivity. J Immunol.

[CR94] Volpe R (1993). Suppressor T lymphocyte dysfunction is important in the pathogenesis of autoimmune thyroid disease: a perspective. Thyroid.

[CR95] Webb DR, Kraig E, Devens BH (1994). Suppressor cells and immunity. Chem Immunol.

[CR96] Xiao C, Rajewsky K (2009). MicroRNA control in the immune system: basic principles. Cell.

[CR97] Zembala M, Asherson GL (1973). Depression of the T cell phenomenon of contact sensitivity by T cells from unresponsive mice. Nature.

[CR98] Zembala M, Asherson GL (1974). T cell suppression of contact sensitivity in the mouse. II. The role of soluble suppressor factor and its interaction with macrophages. Eur J Immunol.

[CR99] Zembala M, Asherson GL, Mayhew B, Krejci J (1975). In vitro absorption and molecular weight of specific T-cell suppressor factor. Nature.

[CR100] Zembala M, Asherson GL, Colizzi V (1982). Hapten-specific T suppressor factor recognizes both hapten and I-J region products on haptenized spleen cells. Nature.

[CR101] Zembala MA, Asherson GL, James BMB, Stein VE, Watkins MC (1982). Anti-haptene T suppressor factor acts through an I-J+, Ly1-2+ , T acceptor cell that releases a nonspecific inhibitor of the transfer of contact sensitivity when exposed to antigen. J Immunol.

